# Chemical Profiling, Molecular Docking, and Mechanistic Anticancer Activity of *Pinus sylvestris* Essential Oil in SH-SY5Y and U-87MG Cells

**DOI:** 10.3390/molecules31030470

**Published:** 2026-01-29

**Authors:** Gökhan Dervişoğlu

**Affiliations:** Department of Molecular Biology and Genetics, Faculty of Arts and Sciences, Bingöl University, Bingöl 12000, Türkiye; gdervisoglu@bingol.edu.tr; Tel.: +90-541-837-77-17

**Keywords:** *Pinus sylvestris* essential oil, GC–MS analysis, molecular docking, ROS, TAS, SH-SY5Y, U-87MG, caspase-dependent apoptosis

## Abstract

*Pinus sylvestris* essential oil (PSEO) has gained increasing interest as a natural anticancer candidate due to its bioactive phytochemical composition and potential to modulate apoptosis-related pathways. In this study, the chemical profile of PSEO was characterized by GC-MS, revealing oxygenated monoterpenes and monoterpene hydrocarbons as dominant constituents. Human brain (U-87MG) and peripheral nervous system (SH-SY5Y) tumor cells were treated with PSEO to evaluate cytotoxicity and mechanistic responses. Cell viability was assessed using the MTT assay, and 24-h IC_50_ values were determined as 47.93 µg/100 µL for U-87MG and 71.63 µg/100 µL for SH-SY5Y, which were subsequently used for all mechanistic analyses. IC_50_ exposure significantly increased intracellular ROS generation while reducing total antioxidant status, indicating oxidative stress-mediated cytotoxicity. Apoptosis-related ELISA assays demonstrated increased caspase-3 and caspase-9 activity, upregulated Bax, decreased Bcl-2, and a lowered Bcl-2/Bax ratio, collectively supporting the activation of the intrinsic mitochondrial apoptosis pathway. Molecular docking provided in silico evidence of favorable binding interactions between selected PSEO-associated ligands and apoptotic targets, consistent with experimentally observed biochemical outcomes. Overall, the findings suggest that PSEO exerts dose- and time-dependent anticancer effects and promotes mitochondrial apoptosis in U-87MG and SH-SY5Y cells, supporting its potential as a natural therapeutic candidate.

## 1. Introduction

Brain-derived malignancies and neuroblastoma-derived tumors represent major clinical challenges due to their high proliferative capacity, invasive behavior, and the development of therapeutic resistance. In glial-origin tumors such as glioblastoma (GBM), standard treatment modalities (including surgical resection, radiotherapy, and chemotherapy) often remain insufficient; high recurrence rates and limited therapeutic outcomes are frequently attributed to tumor heterogeneity, the blood–brain barrier, tumor stem cells, and diverse resistance mechanisms [1,2,3]. Likewise, pediatric and neuroblastoma-derived tumors pose substantial clinical difficulties because of their aggressive tumor biology and poor response to conventional therapies [4].

Consequently, natural compounds with low toxicity profiles and broad-spectrum biological activity have gained increasing attention as potential candidates for novel anticancer strategies [5,6]. Plant-derived essential oils, which are rich in terpenoid and phenolic constituents, represent potent bioactive mixtures capable of exhibiting antioxidant, antiproliferative, antimicrobial, and pro-apoptotic effects [7,8,9,10]. Comprehensive chemical characterization of these oils, together with in vitro assessment of cytotoxicity, oxidative stress, and apoptosis parameters, is essential for elucidating their biological actions. This approach enables the identification of potential therapeutic roles of natural products, particularly those known to modulate the oxidative stress–apoptosis axis in cancer biology.

*Pinus sylvestris* (Scots pine) essential oil (PSEO) is a notable natural product due to its long-standing traditional use and high chemical diversity. Large-scale investigations examining the biological activities of essential oils from *Pinus* species have demonstrated strong antioxidant capacity and significant modulation of oxidative stress mechanisms, including a systematic analysis encompassing 54 pine taxa [11]. Such free-radical scavenging activity is therapeutically relevant in cancer models in which ROS balance plays a key regulatory role in cell proliferation and apoptosis. Additionally, *P. sylvestris* needle extract and essential oil have been reported to exert cytotoxic effects in breast cancer models (MDA-MB-231 and MCF-7), with an IC_50_ value of 29 μg/mL, demonstrating greater cytotoxic potency than the corresponding methanol extract [12]. Furthermore, GC-MS analysis of *Pinus sylvestris* var. *mongolica* needle essential oil has identified numerous terpenoid constituents, and the oil has shown antioxidant, antimicrobial, and breast cancer cell–directed cytotoxic activity [13]. The abundant phenolic, flavonoid, and tannin content of pine needle, bark, and cone fractions further supports the nutraceutical and biomedical potential of this genus, owing to their high antioxidant capacity [8,14].

However, our literature survey revealed no studies evaluating the effects of *P. sylvestris* essential oil in two critical brain tumor models (SH-SY5Y human neuroblastoma and U-87MG human glioblastoma) with respect to cytotoxicity, ROS/oxidative stress, total antioxidant capacity, and apoptosis-related markers such as Caspase-3, Caspase-9, Bax, and Bcl-2. This absence highlights a considerable gap in the literature regarding the effects of PSEO on both neuronal and glial cancer cell models.

This study aims to characterize the chemical composition of PSEO using GC-MS and fatty acid methyl ester (FAME) analyses, predict the interactions of its major constituents with potential target proteins through a molecular docking approach, and comparatively evaluate its cytotoxic, oxidative, and apoptosis-related effects in SH-SY5Y and U-87MG cell lines. By providing a comprehensive assessment of these parameters, the study seeks to elucidate the potential anticancer properties of PSEO in neuroblastoma and glioblastoma models and contribute to the development of natural product-based therapeutic strategies.

## 2. Results

### 2.1. Chemical Composition of Pinus sylvestris Essential Oil Determined by GC–MS Analysis

Steam distillation of fresh needles from *P. sylvestris* yielded 0.6% (*w*/*w*) essential oil (EO), which was colorless, transparent, and had a sharp odor. The PSEO was rich in monoterpenes (95.59%). The phytochemical composition of *Pinus sylvestris* needle essential oil was determined by GC–MS analysis, and twenty-three compounds were identified, representing 100% of the total volatile profile (Table 1). The dominant constituents were bicyclo[3.1.0]hexane, 6-isopropylidene-1-methyl (24.90%), α-terpineol (20.37%), 3-Cyclohexen-1-ol, 1-methyl-4-(1-methylethyl) (9.81%), Cyclohexanol, 1-methyl-4-(1-methylethenyl) (7.03%), Fenchol (6.67%), γ-terpinene (5.15%), terpinen-4-ol (4.52%), and p-Cymene (4.00%). Oxygenated monoterpenes constituted the largest chemical class (57.12%), followed by monoterpene hydrocarbons (38.47%), sesquiterpene hydrocarbons (3.25%), aromatic compounds (0.87%), and non-terpenoid ketones (0.29%).

**Figure 1:**
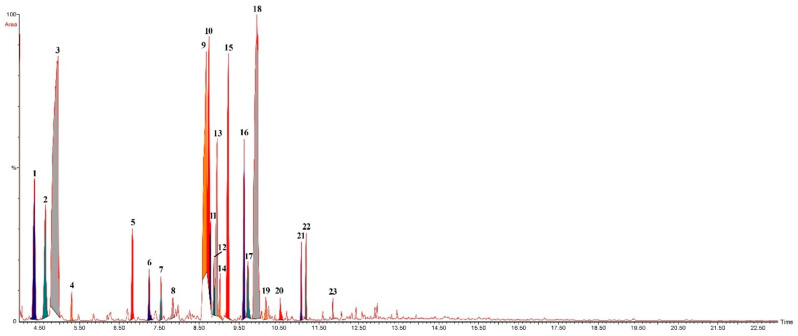
Gas chromatography–mass spectrometry (GC–MS) chromatogram of *Pinus sylvestris* essential oil (PSEO). The analysis was performed to determine the phytochemical composition of the essential oil obtained from fresh *P. sylvestris* needles. Peak numbers correspond to the compounds listed in Table 1. Major monoterpene constituents, including bicyclo[3.1.0]hexane derivatives, α-terpineol, fenchol and terpinen-4-ol, eluted between 8.5 and 10.0 min, whereas monoterpene hydrocarbons such as γ-terpinene and *p*-cymene appeared earlier in the chromatogram (4.3–5.0 min). Low-intensity peaks beyond 11 min represent minor sesquiterpenes and oxygenated monoterpenes. The chromatographic profile supports the compositional distribution shown in Table 1.

The GC–MS chromatogram (Figure 1) exhibited well-resolved peaks, with major signals corresponding to bicyclic monoterpene structures eluting between 8.5 and 10 min, consistent with the high abundance of α-terpineol, fenchol, and terpinen-4-ol. Earlier peaks (4.3–5.0 min) reflected monoterpene hydrocarbons such as γ-terpinene and p-cymene, while later eluting minor constituents appeared as low-intensity signals beyond 11 min. The chromatographic profile fully supported the compositional distribution observed in Table 1.

### 2.2. GC–MS Analysis of FAMEs in PSEO

The chemical composition of FAMEs in *Pinus sylvestris* essential oil (PSEO) was characterized by GC–MS analysis. A total of 15 FAME components were identified, accounting for 100% of the total composition (Table 2). Among these, caproic acid (C6:0) was the predominant constituent, representing 65.02% of the total FAME content, followed by lauric acid (C12:0) at 27.33%. Undecanoic acid (C11:0) (4.98%) and butyric acid (C4:0) (0.99%) were also detected in notable amounts. Minor components, including capric acid (C10:0), caprylic acid (C8:0), palmitic acid (C16:0), trans-elaidic acid (C18:1), myristic acid (C14:0), myristoleic acid (C14:1), tridecanoic acid (C13:0), heptadecanoic acid (C17:0), and cis-10-heptadecenoic acid (C17:1), were present in proportions below 1%. The detailed FAME profile is presented in Table 2.

**Figure 2:**
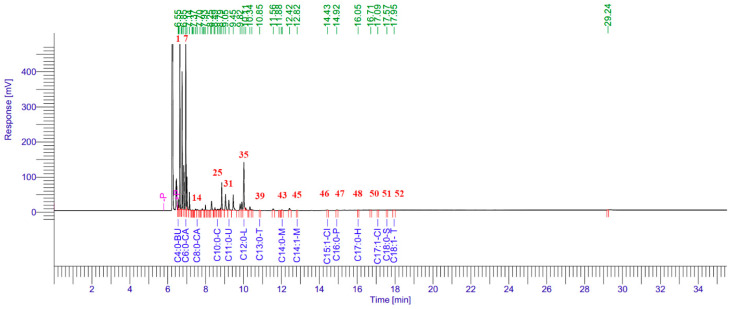
Gas chromatography–mass spectrometry (GC–MS) chromatogram of fatty acid methyl esters (FAMEs) identified in *Pinus sylvestris* essential oil (PSEO). Peak numbers (Peak No.) correspond to the FAME components listed in Table 2. The chromatogram shows two major peaks representing caproic acid methyl ester (C6:0; 65.02%) and lauric acid methyl ester (C12:0; 27.33%), which dominated the total fatty acid profile. Undecanoic acid (C11:0; 4.98%) appeared as a medium-intensity peak, while all remaining FAMEs were detected at <1% abundance and exhibited low-intensity signals between 7–18 min. The chromatographic distribution reflects the relative proportions presented in Table 2.

The GC–MS chromatogram (Figure 2) displayed well-resolved peaks, with major signals corresponding to C6:0 and C12:0, confirming their dominance in the FAME profile. Minor fatty acids showed lower-intensity peaks distributed mainly between 11 and 18 min, consistent with their low abundance in Table 2.

### 2.3. Molecular Docking Analysis

**Figure 3:**
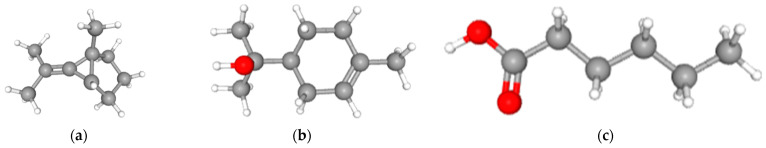
Chemical structures of the ligands used for molecular docking: (**a**) Bicyclo[3.1.0]hexane, 6-isopropylidene-1-methyl-; (**b**) α-terpineol; and (**c**) caproic acid (C6:0).

Molecular docking simulations were carried out to explore the putative binding of selected PSEO-related ligands (Bicyclo[3.1.0]hexane, 6-isopropylidene-1-methyl-; α-terpineol; and caproic acid (C6:0); Figure 3) to apoptosis-associated targets, including caspase-9 (PDB: 1NW9), caspase-3 (PDB: 3KJF), Bax (PDB: 4S0O), and an anti-apoptotic BCL-2 family protein structure (Bcl-xL; PDB: 4C5D), as well as an endonuclease G homology model generated for this study (accession: A29788355) (https://www.rcsb.org/ (accessed on 2 December 2025)). Ligands were selected based on their relative abundance in the chemical profiles of PSEO and to represent major chemical classes detected in the distillate. Specifically, Bicyclo[3.1.0]hexane, 6-isopropylidene-1-methyl- (24.90%) and α-terpineol (20.37%) were among the dominant constituents in the volatile GC–MS profile (Table 1), while caproic acid (C6:0; 65.02%) was the predominant component in the FAME profile (Table 2). Predicted binding affinities (kcal/mol) for all protein–ligand combinations are summarized in Table 3.

**Figure 4:**
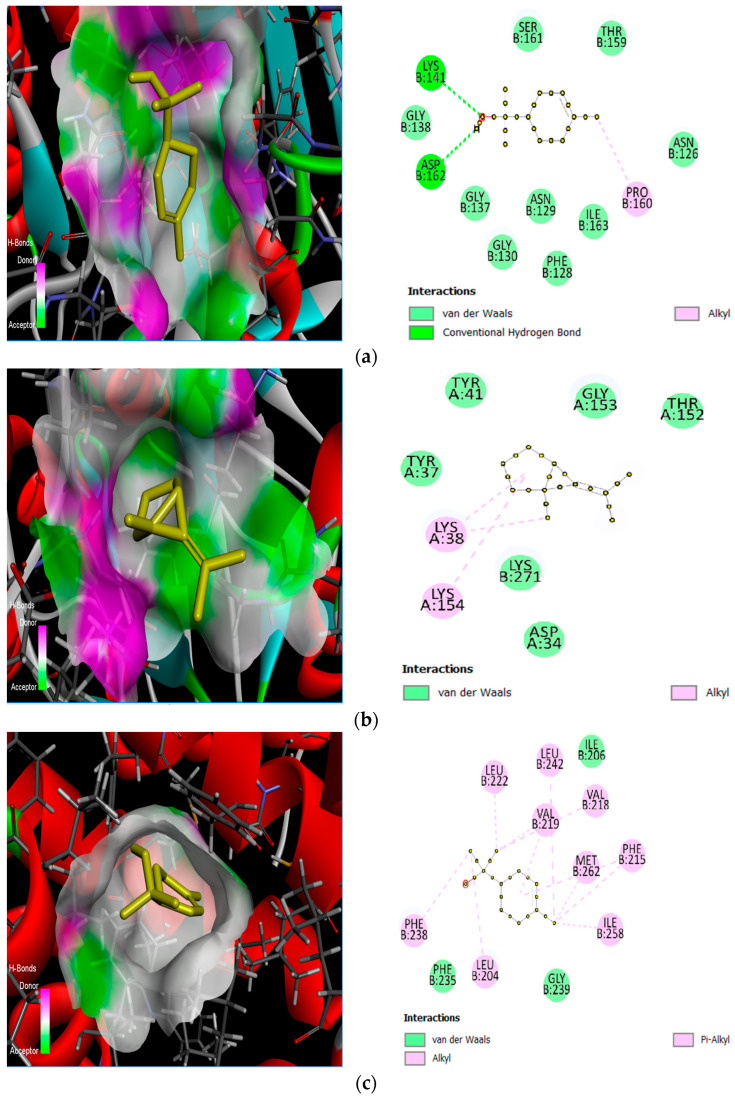

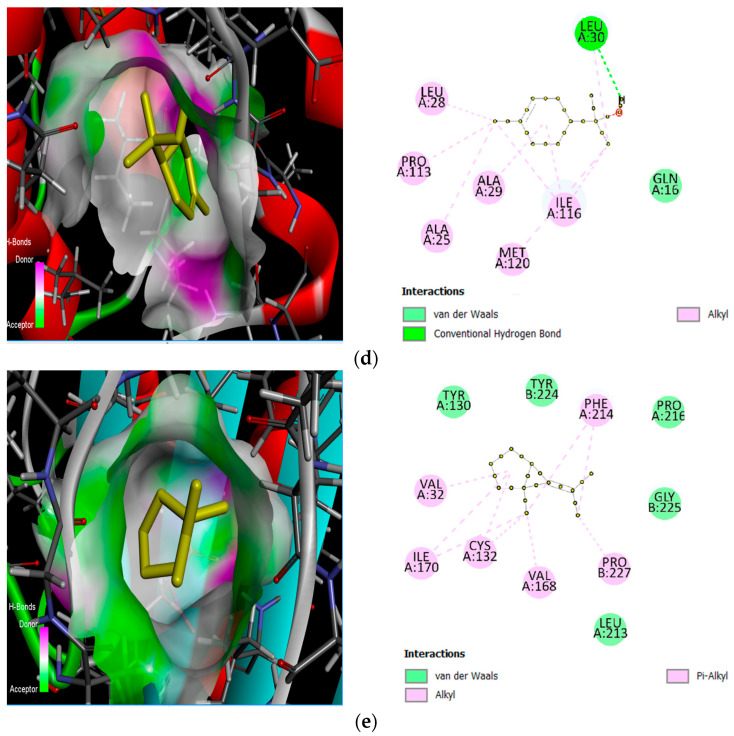
Receptor and ligand molecular docking: (**a**) Caspase-9+α-terpineol (−5.1 kcal/mol); (**b**) Caspase-3+Bicyclo[3.1.0]hexane, 6-isopropylidene-1-methyl- (−4.1 kcal/mol); (**c**) Bax+α-terpineol (−5.8 kcal/mol); (**d**) Bcl-xL (4C5D)+α-terpineol (−6.2 kcal/mol); (**e**) Endonuclease G+Bicyclo[3.1.0]hexane, 6-isopropylidene-1-methyl- (−5.7 kcal/mol).

Among the tested ligands, α-terpineol showed the strongest predicted binding to caspase-9, with a docking score of −5.1 kcal/mol. In the predicted complex, hydrogen bonding was observed between α-terpineol and the side chains of GLY138B and LYS141B (two interactions), together with hydrophobic contacts involving PRO160B and ILE163B (Figure 4a). For caspase-3, the best score was obtained with Bicyclo[3.1.0]hexane, 6-isopropylidene-1-methyl- (−4.1 kcal/mol), supported by hydrophobic interactions with TYR37A, TYR41A, and LYS154A (Figure 4b).

For Bax (4S0O), α-terpineol again yielded the most favorable docking score (−5.8 kcal/mol), and the binding pose was dominated by van der Waals–type contacts with surrounding residues within the predicted binding region (Figure 4c). For Bcl-xL (4C5D), α-terpineol showed the strongest predicted affinity (−6.2 kcal/mol) and formed a hydrogen-bond interaction with Leu30, with additional van der Waals contacts contributing to stabilization (Figure 4d). Finally, in the endonuclease G homology model, Bicyclo[3.1.0]hexane, 6-isopropylidene-1-methyl- exhibited the best score (−5.7 kcal/mol) and showed hydrophobic interactions with VAL32A, TYR130A, VAL168A, ILE170A, TYR224, and PRO227A (Figure 4e).

Overall, docking predicted that α-terpineol consistently achieved the most favorable binding scores against multiple apoptosis-related targets (caspase-9, Bax, and Bcl-xL), whereas the bicyclic ligand showed comparatively stronger affinity toward caspase-3 and endonuclease G in this dataset (Table 3). These computational results support the plausibility of direct ligand–target interactions and provide a structural framework for discussing the apoptosis-linked biochemical changes observed experimentally; however, they should be interpreted as in silico binding hypotheses rather than definitive inhibition evidence.

### 2.4. Cytotoxic Effects of PSEO on U-87MG and SH-SY5Y Cells (MTT Assay)

**Figure 5:**
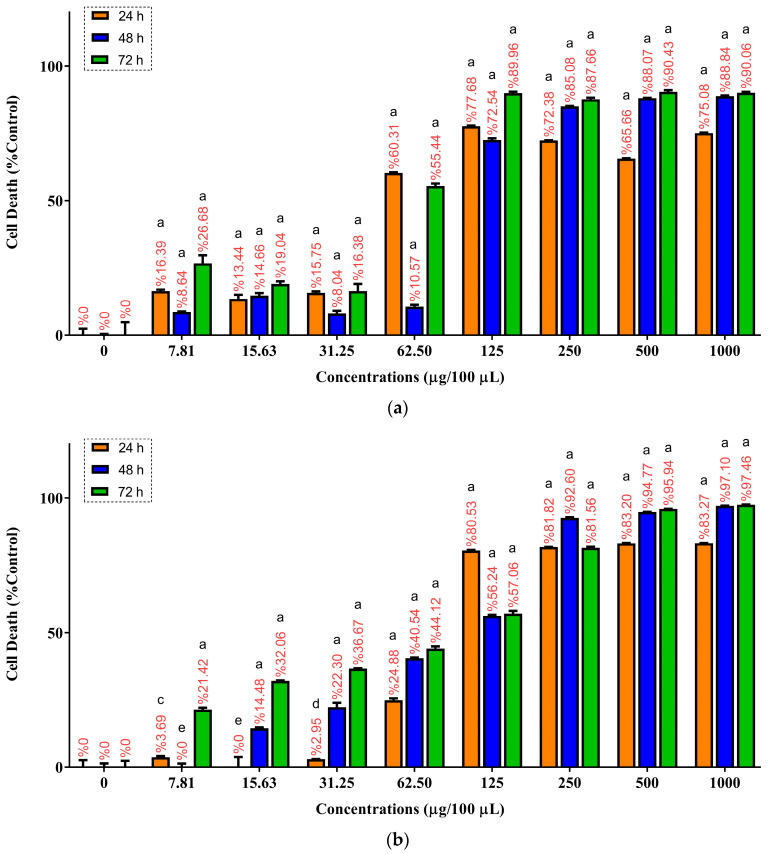
Cytotoxic effects of *Pinus sylvestris* essential oil (PSEO) on U-87MG (**a**) and SH-SY5Y (**b**) cells following 24, 48 and 72 h of exposure. Cells were treated with PSEO at concentrations ranging from 7.81 to 1000 µg/100 µL, and cell death (%) was quantified using the MTT assay. Data are presented as mean ± SD from three independent experiments (*n* = 3) for each concentration and time point. Statistical significance was evaluated relative to the untreated control group (0 µg/100 µL). Different letters above the bars indicate statistical significance levels, where *a* denotes **** *p* < 0.0001, *c* denotes ** *p* < 0.01, *d* denotes * *p* < 0.05, and *e* indicates a non-significant difference (^ns^ *p* > 0.05).

The cytotoxic activity of *Pinus sylvestris* essential oil (PSEO) was evaluated in U-87MG and SH-SY5Y cells following 24, 48 and 72 h of exposure at concentrations ranging from 7.81 to 1000 µg/100 µL. In U-87MG cells, PSEO induced a clear dose-dependent reduction in cell viability at all incubation times (Figure 5a). After 24 h of treatment, cell death increased from 16.39% at 7.81 µg/100 µL to 77.68% at 125 µg/100 µL and remained above 65% at 250–1000 µg/100 µL. At 48 h, the cytotoxic response was less pronounced at lower concentrations, although cell death was markedly increased at 250–1000 µg/100 µL (85.08–88.84%). After 72 h, PSEO again exhibited strong cytotoxicity, with cell death rising to 89.96% at 125 µg/100 µL and above 90% at concentrations ≥500 µg/100 µL. All concentrations tested (7.81–1000 µg/100 µL) produced extremely significant cytotoxicity compared with the untreated control at all incubation times (**** *p* < 0.0001). The IC_50_ values for U-87MG cells were calculated as 47.93, 100.88, and 61.15 µg/100 µL for 24, 48 and 72 h, respectively.

In SH-SY5Y cells, PSEO also increased cell death in a dose- and time-dependent manner (Figure 5b). At 24 h, low-dose exposure produced minimal cytotoxicity, with cell death measured as 3.69% at 7.81 µg/100 µL (** *p* < 0.01), 0.00% at 15.63 µg/100 µL (^ns^ *p* > 0.05), and 2.95% at 31.25 µg/100 µL (* *p* < 0.05). Higher concentrations caused a sharp transition to pronounced cytotoxicity, with 80.53% and 81.82% cell death at 125 and 250 µg/100 µL, respectively, all of which were extremely significant compared with the control (**** *p* < 0.0001). After 48 h, cytotoxicity remained extremely significant across all concentrations except 7.81 µg/100 µL (^ns^ *p* > 0.05), with cell death values increasing to 92.60% and 94.77% at 250 and 500 µg/100 µL, respectively (**** *p* < 0.0001). By 72 h, every tested concentration (7.81–1000 µg/100 µL) induced extremely significant cytotoxicity relative to the control (**** *p* < 0.0001), with the highest doses (500–1000 µg/100 µL) exceeding 95% cell death. The IC_50_ values for SH-SY5Y cells were determined to be 71.63, 86.30 and 192.66 µg/100 µL for 24, 48 and 72 h, respectively. The raw optical density (OD) values used for MTT-based calculations in U-87MG and SH-SY5Y cells are provided in the Supplementary Materials (Tables S1 and S2).

Based on these results, the 24-h IC_50_ concentration determined for each cell line was selected for all subsequent biochemical assays, including ROS, TAS, caspase activity, and Bcl-2/Bax analyses. This ensured that the mechanistic studies were performed under comparable cytotoxic conditions.

### 2.5. Intracellular ROS Levels in U-87MG and SH-SY5Y Cells

**Figure 6:**
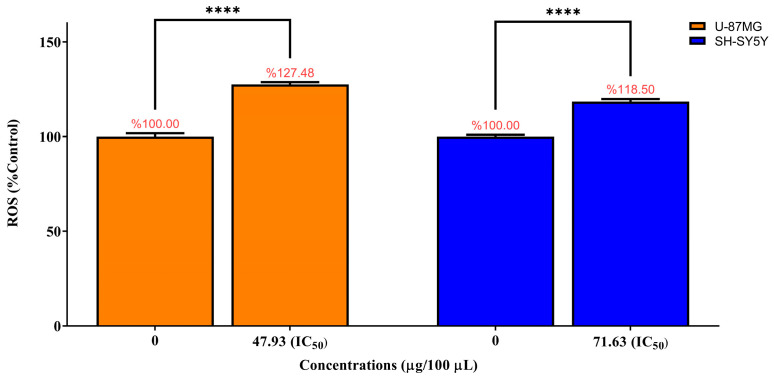
Intracellular reactive oxygen species (ROS) levels in U-87MG and SH-SY5Y cells following 24 h exposure to *Pinus sylvestris* essential oil (PSEO) at their respective IC_50_ concentrations. ROS production was quantified using the DCFH-DA fluorescence assay and expressed as percentage of the untreated control group (0 µg/100 µL PSEO; % of control). Data represent mean ± SD from three independent experiments (*n* = 3). PSEO treatment significantly increased ROS generation in both tumor cell lines compared with their respective untreated controls (**** *p* < 0.0001), indicating a strong oxidative response induced by IC_50_ exposure.

Intracellular ROS levels were quantified after 24 h exposure of U-87MG and SH-SY5Y cells to PSEO at their respective 24-h IC_50_ concentrations (47.93 µg/100 µL for U-87MG and 71.63 µg/100 µL for SH-SY5Y). As shown in Figure 6, PSEO induced a pronounced oxidative response in both tumor cell lines. In U-87MG cells, ROS levels increased to 127.48 ± 1.19% of control, corresponding to a 1.27-fold elevation relative to untreated cells (0 µg/100 µL). Similarly, SH-SY5Y cells exhibited a significant rise in ROS production, reaching 118.50 ± 1.38% of control (1.18-fold increase). Statistical analysis confirmed that IC_50_ treatment caused a highly significant increase in intracellular ROS generation compared with the untreated control group (0 µg/100 µL; **** *p* < 0.0001). The raw fluorescence intensity values underlying the ROS assay are provided in the Supplementary Materials (Table S3).

These findings demonstrate that PSEO triggers a strong oxidative stress response in both central and peripheral nervous system–derived tumor cells. The observed ROS elevation is consistent with the downstream activation of mitochondrial apoptotic signaling, including caspase-3 and caspase-9 activation and a shift in the Bcl-2/Bax ratio, suggesting that oxidative stress may contribute substantially to PSEO-induced cytotoxicity.

### 2.6. Total Antioxidant Status (TAS) in U-87MG and SH-SY5Y Cells

**Figure 7:**
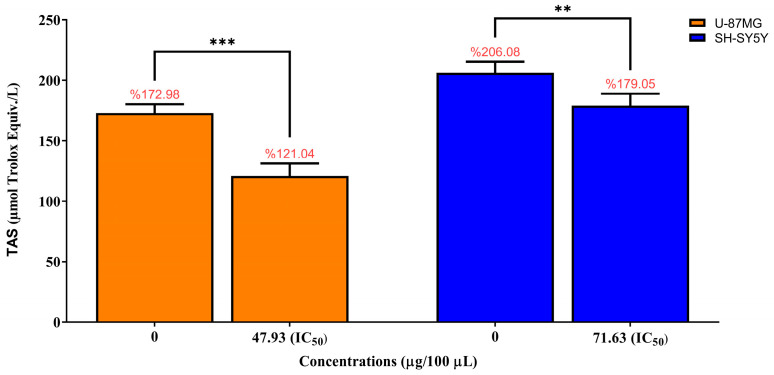
Total antioxidant status (TAS) in U-87MG and SH-SY5Y cells following 24 h exposure to *Pinus sylvestris* essential oil (PSEO) at their respective IC_50_ concentrations. TAS levels were quantified using a commercial TAS assay kit and expressed as µmol Trolox equivalents per liter (µmol Trolox/L). Data represent mean ± SD from three independent experiments (*n* = 3). Compared with the untreated control group (0 µg/100 µL PSEO), PSEO treatment significantly reduced TAS levels in both U-87MG (*** *p* < 0.001) and SH-SY5Y (** *p* < 0.01) cells, indicating a decline in total antioxidant capacity upon IC_50_ exposure.

Total antioxidant status (TAS) was quantified in U-87MG and SH-SY5Y cells following 24 h exposure to *Pinus sylvestris* essential oil (PSEO) at their respective IC_50_ concentrations (47.93 µg/100 µL for U-87MG and 71.63 µg/100 µL for SH-SY5Y). As shown in Figure 7, TAS levels in U-87MG cells markedly decreased from 172.98 ± 7.29 µmol Trolox/L in untreated controls to 121.04 ± 10.20 µmol Trolox/L after IC_50_ treatment, representing a statistically significant reduction (*** *p* < 0.001). Similarly, SH-SY5Y cells exhibited a significant decrease in antioxidant capacity, declining from 206.08 ± 9.28 µmol Trolox/L in the control group to 179.05 ± 9.89 µmol Trolox/L following exposure to the 24-h IC_50_ dose (71.63 µg/100 µL; ** *p* < 0.01). The raw absorbance values used for TAS calculations are provided in the Supplementary Materials (Table S4).

These findings demonstrate that PSEO reduces intracellular total antioxidant capacity in both tumor cell lines, supporting the notion that PSEO-induced cytotoxicity is associated with a shift toward a pro-oxidant cellular environment, consistent with the elevated ROS levels observed under IC_50_ treatment.

### 2.7. Apoptosis-Related Protein Levels in U-87MG and SH-SY5Y Cells

**Figure 8:**
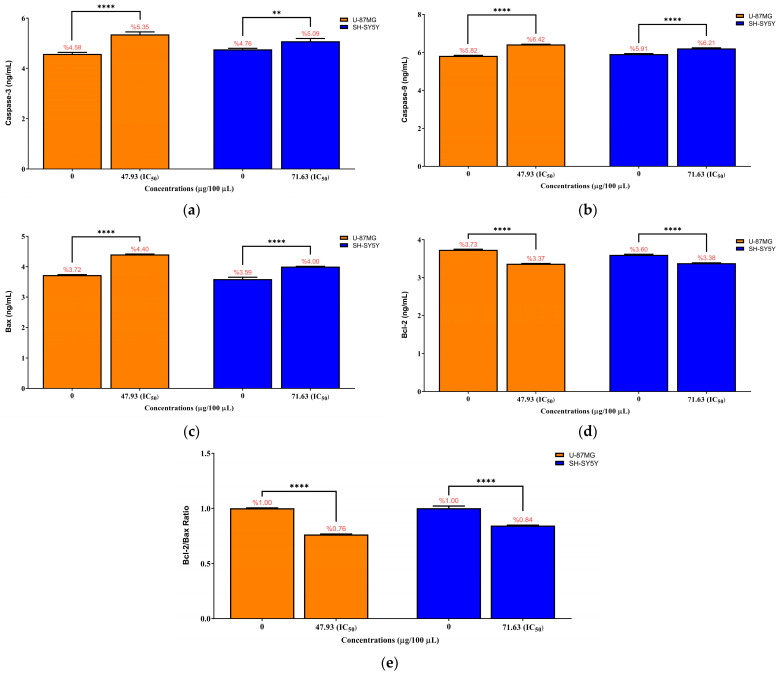
Effects of *Pinus sylvestris* essential oil (PSEO) on apoptosis-related protein levels in U-87MG and SH-SY5Y cells following 24 h exposure at their respective IC_50_ concentrations (47.93 and 71.63 µg/100 µL, respectively). (**a**) Caspase-3, (**b**) caspase-9, (**c**) Bax, (**d**) Bcl-2 protein levels, and (**e**) Bcl-2/Bax ratio were quantified using ELISA. Data are expressed as mean ± SD from three independent experiments (*n* = 3). PSEO significantly increased caspase-3, caspase-9, and Bax levels while reducing Bcl-2 levels in both cell lines, leading to a marked decrease in the Bcl-2/Bax ratio. Statistical comparisons were performed relative to the untreated control group. Statistical significance was evaluated relative to the untreated control group (0 µg/100 µL PSEO). Significance levels are indicated by brackets in the figure as follows: (** *p* < 0.01) and (**** *p* < 0.0001).

Apoptosis-associated protein levels were quantified in U-87MG and SH-SY5Y cells after 24 h exposure to PSEO at their respective IC_50_ concentrations (47.93 µg/100 µL for U-87MG and 71.63 µg/100 µL for SH-SY5Y). As shown in Figure 8a, PSEO significantly increased caspase-3 levels in both cell lines. In U-87MG cells, caspase-3 increased from 4.578 ± 0.059 ng/mL in the control group to 5.353 ± 0.099 ng/mL after treatment (**** *p* < 0.0001), whereas SH-SY5Y cells showed an increase from 4.756 ± 0.039 ng/mL to 5.086 ± 0.104 ng/mL (** *p* < 0.01). Caspase-9 levels (Figure 8b) also increased significantly, rising from 5.817 ± 0.026 ng/mL to 6.423 ± 0.007 ng/mL in U-87MG cells and from 5.913 ± 0.024 ng/mL to 6.210 ± 0.029 ng/mL in SH-SY5Y cells (both **** *p* < 0.0001).

Pro-apoptotic Bax levels (Figure 8c) increased markedly following IC_50_ exposure (**** *p* < 0.0001), increasing from 3.724 ± 0.014 ng/mL to 4.404 ± 0.013 ng/mL in U-87MG cells and from 3.591 ± 0.062 ng/mL to 4.003 ± 0.009 ng/mL in SH-SY5Y cells. In contrast, anti-apoptotic Bcl-2 levels (Figure 8d) decreased significantly (**** *p* < 0.0001), falling from 3.730 ± 0.017 ng/mL to 3.366 ± 0.007 ng/mL in U-87MG cells and from 3.597 ± 0.017 ng/mL to 3.381 ± 0.010 ng/mL in SH-SY5Y cells.

Correspondingly, the Bcl-2/Bax ratio (Figure 8e) decreased substantially, declining from 1.002 to 0.764 in U-87MG cells and 1.002 to 0.845 in SH-SY5Y cells (**** *p* < 0.0001), indicating a clear shift toward a pro-apoptotic intracellular environment. This coordinated pattern of caspase activation, Bax upregulation, and Bcl-2 suppression demonstrates that PSEO promotes apoptosis in both tumor cell lines through a mitochondria-dependent pathway. The raw ELISA absorbance values (OD450) and standard curve data used for quantification are provided in the Supplementary Materials (Tables S5–S8).

## 3. Discussion

In this study, the cytotoxic potential of *Pinus sylvestris* essential oil (PSEO) was evaluated in U-87MG glioblastoma and SH-SY5Y neuroblastoma cell lines by integrating cell viability with oxidative stress (ROS, TAS) and apoptosis-associated protein markers (caspase-3, caspase-9, Bcl-2, Bax, and Bcl-2/Bax ratio). Overall, the findings support a mechanism in which PSEO perturbs cellular redox homeostasis and promotes mitochondria-associated apoptotic signaling. This integrated interpretation is consistent with current understanding that cancer cells often operate under elevated intrinsic oxidative stress, and a further ROS burden can exceed antioxidant buffering and favor regulated cell death programs [15,16].

Importantly, the GC–MS compositional profile of PSEO indicates a terpene-rich essential oil matrix, including fractions typically described as monoterpene hydrocarbons, oxygenated monoterpenes, sesquiterpene hydrocarbons, and related aromatics or non-terpenoid components. Without attributing activity to any single compound, this class-level profile aligns with mechanistic trends widely reported for essential oils in cancer models, where terpene-dominant mixtures frequently induce redox imbalance, mitochondrial dysfunction, and apoptosis-linked signaling [17]. In line with this broader framework, Pinus-derived essential oils have been reported to drive ROS-associated, caspase-linked apoptosis in cancer cells, supporting the plausibility that a terpene-rich Pinus oil can generate the combined oxidative and apoptotic signature observed here [18].

In plant-derived preparations, the extraction method and solvent/vehicle selection can markedly influence both the chemical composition and the resulting biological outcomes. Steam distillation is designed to enrich volatile and predominantly hydrophobic constituents in the essential oil fraction, whereas classical solvent-based extraction approaches (e.g., alcoholic or aqueous solvents) may yield substantially different profiles by recovering more polar and/or non-volatile compounds. Therefore, differences in solvent polarity and extraction conditions can shift the relative abundance of chemical classes and may lead to divergent cellular responses. In the present work, all cell-based assays were performed using the essential oil fraction obtained by distillation, and DMSO was employed only as a vehicle to ensure homogeneous dispersion and dosing of hydrophobic constituents. The final DMSO concentration was kept constant and below 0.5% (*v*/*v*) in all conditions with matched vehicle controls to minimize solvent-related confounding effects [19,20,21,22,23].

Computational analysis (in silico) was performed separately from the experimental assays and is presented here strictly as hypothesis-generating evidence, not as experimentally validated target modulation. To complement the biochemical findings, molecular docking was incorporated to explore whether selected PSEO-associated ligands could theoretically interact with apoptosis-related targets (caspase-9, caspase-3, Bax, Bcl-2) and Endonuclease G. The workflow, which was based on Protein Data Bank (PDB) structures and SWISS-MODEL homology models, followed by AutoDock Vina (v1.1.2) scoring within the UCSF Chimera environment, represents a widely accepted strategy for hypothesis generation rather than functional proof. Docking is recognized to estimate relative affinity and plausible binding orientations, but the interpretation of scores must consider methodological constraints such as rigid-receptor assumptions, limited conformational sampling, and simplified scoring functions [24,25]. These limitations are well established in the literature, particularly for small hydrophobic ligands whose binding energies often fall within moderate ranges that cannot alone confirm inhibition or activation [26,27]. Within this framework, α-terpineol demonstrated comparatively favorable binding energies toward caspase-9 and Bcl-2, whereas Bicyclo[3.1.0]hexane, 6-isopropylidene-1-methyl- ligands showed stronger affinity toward caspase-3 and Endonuclease G. These in silico observations are directionally consistent with the experimentally observed intrinsic apoptosis profile, characterized by increased caspase-9 and caspase-3 activity, elevated Bax expression, reduced Bcl-2 levels, and a decreased Bcl-2/Bax ratio. Thus, while the docking results do not establish causality, they present a coherent structural hypothesis that PSEO compounds may engage apoptosis-associated proteins through non-covalent interactions. Although the selected compounds represent the most abundant constituents of the essential oil, the biological effects observed for Pinus sylvestris essential oil reflect the combined action of multiple components. Therefore, synergistic or antagonistic interactions among constituents cannot be excluded [28]. Nevertheless, abundance-based selection does not imply that these ligands solely explain the experimentally observed bioactivity. Essential oils act as complex phytochemical mixtures (“phytocomplexes”), and their cellular effects may arise from additive, synergistic, or even antagonistic interactions among major and minor constituents [7,17,28]. Therefore, the docking outcomes should not be interpreted as direct validation that a single constituent drives the cytotoxic phenotype, but rather as a focused computational exploration of plausible target engagement by representative, high-abundance molecules. In this context, the molecular docking results should be interpreted cautiously and considered as supportive, hypothesis-generating mechanistic insights rather than definitive explanations of the extract’s biological activity [17,29]. This limitation is inherent to studies investigating complex natural mixtures such as essential oils and has been widely acknowledged in the literature [7,17,28,29]. Collectively, the in vitro assays provide experimental evidence for redox imbalance and intrinsic apoptosis-linked signaling, whereas the docking results should be interpreted only as supportive, hypothesis-generating predictions that require direct experimental validation.

The MTT assay demonstrated that PSEO decreased cell viability in both cell lines across 24, 48, and 72 h in a concentration-dependent manner. For mechanistic analyses, the 24 h IC_50_ concentration determined for each cell line was used in all subsequent biochemical assays, providing a standardized exposure condition that captures early signaling changes under comparable cytotoxic stress. This single-dose IC_50_ strategy is frequently used in essential-oil anticancer literature to reduce confounding from extensive late-stage cell loss while enabling direct comparison of mechanistic readouts [17]. However, we acknowledge that including additional sub-IC_50_ and supra-IC_50_ concentrations would further strengthen the dose–response relationship and mechanistic causality between PSEO exposure and apoptosis-related signaling. Future studies should therefore incorporate multi-dose validation to better define concentration-dependent shifts in ROS/TAS balance and intrinsic apoptosis markers.

Consistent with a redox-mediated mechanism, IC_50_ exposure significantly increased intracellular ROS levels in both U-87MG and SH-SY5Y cells. This finding is compatible with the concept that ROS can act as a pro-death trigger when oxidative pressure surpasses cellular tolerance thresholds, particularly in malignant cells with already altered redox regulation [15,16]. A comparable directionality, namely ROS elevation accompanying cytotoxicity, is repeatedly emphasized as a common mechanistic route in essential oil–mediated anticancer effects [17]. Although increased ROS levels were consistently observed following PSEO exposure, we cannot definitively conclude causality in the absence of ROS-scavenger/rescue experiments (e.g., NAC). Future studies incorporating antioxidant pretreatment designs will be important to determine whether ROS elevation is an upstream trigger that drives apoptosis-related signaling or a downstream consequence of cytotoxicity.

In parallel, TAS analysis showed a reduction in total antioxidant capacity after IC_50_ exposure, reinforcing a shift toward a pro-oxidant intracellular environment. The TAS assay principle applied here, based on ABTS radical decolorization and Trolox-equivalent calibration, is consistent with the established methodology described by Erel and with the Rel Assay Diagnostics TAS kit principle (absorbance change at 660 nm reflecting total antioxidant status) [30]. Together with the ROS increase, the TAS decrease supports the interpretation that PSEO disrupts redox balance, a mechanistic pattern also commonly reported across essential oils in cancer models [17].

Apoptosis-associated protein profiling further indicated activation of intrinsic apoptosis signaling. Both caspase-9 and caspase-3 levels were increased following IC_50_ exposure, consistent with initiation and execution phases of mitochondria-associated caspase cascades. This interpretation aligns with canonical apoptosome-linked activation models in which caspase-9 acts as an initiator caspase downstream of mitochondrial permeabilization and supports activation of executioner caspases such as caspase-3 [31,32].

Concordantly, Bax was increased and Bcl-2 was decreased in both cell lines, resulting in a reduced Bcl-2/Bax ratio. The observed shift toward higher Bax with lower Bcl-2 supports enhanced susceptibility to intrinsic apoptosis, because the balance of BCL-2 family proteins regulates mitochondrial outer membrane permeabilization and thereby sets the apoptotic threshold [33,34]. The overall pattern, namely ROS elevation, reduced antioxidant capacity, caspase activation, Bax upregulation, and Bcl-2 suppression, is consistent with mechanistic signatures frequently described for essential oil–induced apoptosis and has also been reported for Pinus-related essential oils in cancer cell contexts [18]. However, complementary apoptosis assays (e.g., Annexin V/PI staining, mitochondrial membrane potential analysis, and/or DNA fragmentation assays) would further strengthen pathway confirmation and help distinguish apoptosis from other potential cell death mechanisms; these validations will be addressed in future studies.

Taken together, the present findings provide convergent evidence that PSEO exerts cytotoxic effects in U-87MG and SH-SY5Y cells and promotes a redox-associated, mitochondria-linked apoptotic response, as supported by the coordinated ROS and TAS shift and by caspase and Bcl-2 family modulation. This mechanism-level interpretation is compatible with broader literature proposing that redox targeting can selectively pressure malignant cells and that essential oils may act as multi-target mixtures capable of engaging oxidative and apoptotic pathways [15,16].

Although essential oils are widely investigated as bioactive mixtures, safety considerations are particularly important because volatile constituents can act as irritants and may exhibit non-selective cytotoxicity at higher concentrations [29,35,36]. Indeed, essential oils and individual monoterpenes/alcohols have been reported to cause irritation and toxicity depending on the route, dose, and exposure duration, and their hydrophobic nature can contribute to membrane-related cytotoxic effects [36]. Therefore, the present findings should be interpreted as proof-of-concept evidence of in vitro anticancer potential rather than a direct indication of safe therapeutic applicability in humans [29,35]. Importantly, translating in vitro cytotoxicity data to a clinically relevant context has inherent limitations. Cell culture systems do not fully recapitulate the complexity of human exposure, including absorption, distribution, metabolism, and clearance, as well as tissue barriers and protein binding that determine free (bioavailable) concentrations in vivo [37,38,39]. In the case of essential oils, translation is further complicated by their volatility and formulation-dependent bioavailability, meaning that achievable tissue exposures may differ substantially from nominal in vitro concentrations. Therefore, in vivo pharmacokinetic/bioavailability studies and delivery optimization (e.g., nanoformulations or encapsulation strategies) are essential prerequisites before any therapeutic inference can be made. Quantitative in vitro-to-in vivo extrapolation (QIVIVE) supported by physiologically based kinetic (PBK) modelling is increasingly recommended to relate in vitro effect concentrations to realistic external doses and tissue-level exposures; however, such modelling requires additional kinetic and exposure data that were beyond the scope of the present study. Moreover, essential oils are complex natural mixtures with batch-to-batch variability, and safety assessment for human use typically requires standardized composition and dedicated toxicological testing (e.g., irritation/sensitization, systemic toxicity, and in vivo tolerability studies), together with appropriate formulation strategies to reduce local irritation and improve selective delivery [36,40,41].

Finally, since our work focused on tumor cell responses, future studies should include non-tumorigenic (normal) human cell models to assess selectivity indices, and should be complemented by in vivo evaluations to better define therapeutic windows and safety margins.

## 4. Materials and Methods

### 4.1. Plant Material

Pine (*Pinus sylvestris*) branches were collected from the Çevirme region of Genç district, Bingöl province, at an altitude of 1200 m in July 2025. Plant material was botanically identified by Dr. Abdurrahim Çetin (Department of Molecular Biology and Genetics, Faculty of Arts and Sciences, Bingöl University, Türkiye). It has been registered with the voucher specimen No. BIN13444 (*Pinus sylvestris*) in the Bingöl University Herbarium Research Laboratory. Fresh pine needles were collected from its pine branches, dried at room temperature (25 °C) with ventilation, and cut into small pieces.

### 4.2. Essential Oil Isolation

The essential oil was isolated from dried small pine needles by steam distillation as described in Aloui et al. [42]. This process was conducted in triplicate over a duration of 3 h, utilizing 500 g of dried small pine needles, which were put into 1 L of distilled water. After extraction, the *Pinus sylvestris* essential oil (PSEO) was dried using anhydrous sodium sulfate and stored at 4 °C until all analyses were performed. After distillation, the essential oil phase was physically separated from the aqueous distillate (hydrosol). The hydrosol fraction was not used in any of the cell-based assays; all biological experiments were performed using the essential oil fraction (PSEO).

### 4.3. Essential Oil Sample Preparation for GC-MS Analysis

A PSEO sample was prepared before GC-MS analysis. For this purpose, 1 mL of isolated PSEO was put into a test tube and 4 mL of hexane was added to it. The mixture was vortexed and centrifuged for 5 min. The supernatant was collected and filtered through a 0.22 µm filter. The filtrate was placed in a GC vial to perform GC-MS analysis. This hexane-dilution step was applied exclusively for GC–MS injection and was not used for any cell-based experiments.

### 4.4. GC-MS Analysis

The PSEO was analyzed by GC-MS using a PerkinElmer Clarus 690 (PerkinElmer, Waltham, MA, USA) gas chromatograph combined with a PerkinElmer Clarus SQ 8T (PerkinElmer, Waltham, MA, USA) mass spectrometer system, powered by TurbaMass Ver. 6.1.2 software. A flame ionization detector (FID) equipped with Elite-WAX capillary column (PerkinElmer, Waltham, MA, USA) (30 m × 0.25 mm I.D. × 0.25 µm film thickness) was used with helium as carrier gas. GC oven temperature was kept at 70 °C for 1 min and programmed to 220 °C at a rate of 2 °C/min, then kept constant at 220 °C for 10 min. The split ratio was adjusted to 10:1. The injector and detector temperatures were 240 °C. MS were taken at 70 eV. Mass range was from *m*/*z* 10–400. A library search was carried out using the NIST Mass Spectral Library. Relative percentage amounts were calculated from the total ion chromatogram (TIC) by the computer.

### 4.5. Methyl Esterification of Fatty Acids

In order to determine the fatty acid profile of PSEO by GC/MS analysis, the PSEO sample was first derivatized according to the base-catalyzed methyl esterification (alkaline methylation) method defined in standard methodologies such as AOAC 969.33 [43], AOCS Ce 2-66 [44], or ISO 5509 [45]. For this purpose, 6 mL of hexane was added to the 200 µL PSEO sample placed in the test tube, and vortexed to ensure the extraction of fatty acids from the PSEO sample. Then, 200 µL of 2 N KOH was added and vortexed to convert fatty acids into methyl esters (FAME). The mixture was centrifuged for 5 min. In the phase separation that occurred after the reaction, the upper phase (hexane + FAMEs) was taken as supernatant, filtered with a 0.22 µm filter and used for GC/MS analysis by placing it in a GC-FID vial.

### 4.6. GC Conditions for the FAME

The fatty acid profile was analyzed by GC-MS using a PerkinElmer Clarus 690 gas chromatograph combined with a PerkinElmer Clarus SQ 8T mass spectrometer system, powered by TurbaMass Ver. 6.1.2 software. A flame ionization detector (FID) equipped with Elite-WAX capillary column (30 m × 0.25 mm I.D. × 0.25 µm film thickness) was used with helium as carrier gas. GC oven temperature was programmed at 250 °C, with three ramps: first ramping at 10 °C/min, held for 5 min; second ramping at 5 °C/min, held for 5 min; third ramping at 5 °C/min, held for 8 min. The split ratio was adjusted to 20:1. The injector and detector temperatures were 250 °C. F.A.M.E. Mix, C_4_–C_24_ (mixture of 37 FAME, Bellefonte, PA, USA) reference standard was used in the validation procedure. Relative percentage amounts were calculated from the total ion chromatogram (TIC) by the computer.

### 4.7. Molecular Docking

The compounds selected for molecular docking were chosen based on their relative abundance in the essential oil as determined by GC–MS and FAME analyses. Compounds present at the highest proportions were prioritized, as these constituents are more likely to contribute to the biological effects of the extract. In addition, the selection was supported by previous literature reporting biological activity of these compounds in cell culture models. Accordingly, molecular docking was employed as a supportive, hypothesis-generating tool rather than as direct evidence of causality. Molecular docking was performed to explore the potential interactions between apoptosis-related targets (caspase-9, caspase-3, Bax, Bcl-2 family protein, and endonuclease G) and selected compounds identified from *Pinus sylvestris* essential oil (PSEO). The available three-dimensional structures of the target proteins were retrieved from the Protein Data Bank (PDB) (caspase-9, PDB: 1NW9; caspase-3, PDB: 3KJF; Bax, PDB: 4S0O; and the anti-apoptotic BCL-2 family structure used in this study, PDB: 4C5D) (https://www.rcsb.org/). A homology model was generated using the SWISS-MODEL server based on the corresponding protein sequence (accession as used in this work: A29788355), because an experimental structure was not available for endonuclease G [46]. Ligand structures were obtained from PubChem [47]. Docking simulations were conducted using AutoDock Vina (v1.1.2) implemented in UCSF Chimera (v1.17.3) to estimate binding affinities of the selected ligands (Bicyclo[3.1.0]hexane, 6-isopropylidene-1-methyl-; α-Terpineol; and caproic acid (C6:0)) toward the target proteins [48,49]. Protein–ligand hydrogen-bonding and interaction patterns were inspected in UCSF Chimera, and 2D/3D interaction visualizations were additionally generated using BIOVIA Discovery Studio Visualizer (2024) [50]. For each target, the docking search space was centered on the binding region using the following grid box center coordinates (x, y, z): caspase-9 (40.08, 10.04, 101.699), caspase-3 (6.436, 2.479, 13.046), Bax (24.339, 3.6167, 12.48), BCL-2 family protein (0.3442375, −21.2879, −42.3977), and endonuclease G (15.00, 2.197, 71.23); all remaining parameters were kept at default settings of the Chimera–Vina workflow. The docking outputs were subsequently used to support interpretation of possible interaction modes of the selected high-abundance PSEO constituents with apoptosis-pathway targets.

### 4.8. Cell Culture

In this study, human central nervous system cancer U-87 MG (ATCC^®^ HTB-14™) and human peripheral nervous system tumor SH-SY5Y (ATCC^®^ CRL-2266™) cell lines were obtained from the American Type Culture Collection (ATCC; Manassas, VA, USA) and used for cytotoxicity, oxidative stress, antioxidant capacity, and apoptosis-related analyses of PSEO. The U-87MG and SH-SY5Y cell lines were cultured and propagated according to previously published protocols with minor modifications [51,52,53,54]. Briefly, 1 × 10^6^ U-87MG or SH-SY5Y cells were seeded into T25 flasks containing 4 mL of complete medium and maintained at 37 °C in a humidified incubator with 5% CO_2_ until approximately 80% confluence was reached. The complete medium consisted of RPMI 1640 base medium supplemented with 10% fetal bovine serum and 0.5% penicillin-streptomycin antibiotic solution. The cells were subcultured every three days. All experiments were initiated when the cells reached approximately 80% confluence.

### 4.9. Essential Oil Treatment for Cytotoxicity and Biochemical Assays

For all experimental procedures, U-87 MG and SH-SY5Y cells were seeded into appropriate culture vessels depending on the assay: 1.5 × 10^4^ cells per well were plated in 96-well plates for the MTT cytotoxicity and TAS analyses, 6 × 10^5^ cells per well were seeded into 6-well plates for intracellular ROS measurements, and 2 × 10^6^ cells were cultured in T-25 flasks for apoptosis-related protein quantification (caspase-3, caspase-9, Bcl-2 and Bax). Following overnight attachment at 37 °C with 5% CO_2_, the culture medium was replaced with fresh complete medium containing PSEO. For all cell-based assays, “PSEO” refers exclusively to the essential oil fraction obtained after distillation and drying (not the aqueous distillate/hydrosol). PSEO stock solutions were prepared in DMSO, and the final DMSO concentration in all treatment media was kept below 0.5% to avoid solvent-induced cytotoxicity. For the MTT assay, cells were treated with a range of PSEO concentrations (7.81–1000 µg/100 µL) and incubated for 24, 48 or 72 h. For all biochemical analyses (ROS, TAS, caspase-3, caspase-9, Bcl-2 and Bax), cells were treated with PSEO at the 24-h IC_50_ concentration previously determined for each cell line, while control groups received medium containing an equivalent amount of DMSO (<0.5%, *v*/*v*). After the 24-h treatment period, cells or culture supernatants were processed according to the requirements of each assay, as described in the corresponding methodological sections. All experiments were performed in three independent biological replicates.

### 4.10. MTT Cell Viability Assay

MTT cell viability assay to determine the cytotoxic activity of PSEO on U-87 MG and SH-SY5Y cells was performed according to the protocol described in the study of Kemal Alp et al. [55]. For this purpose, 5 mg of MTT reagent powder was dissolved in 1 mL of PBS and passed through a 0.22 µm filter, and then 9 mL of complete medium was added to prepare a 10% (*w*/*v*) MTT reagent solution. At the end of the specified 24-, 48- and 72-h incubation periods, the existing medium in each well of the 96-well plate was discarded and then replaced with 100 µL 10% (*w*/*v*) MTT reagent solution. In order for living cells to metabolize tetrazolium salts to formazan crystals by mitochondrial dehydrogenase enzymes, cells were incubated for 4 h at 37 °C in a 5% CO_2_ incubator. At the end of the incubation period, the MTT reagent solution in each well of the 96-well plate was removed, 100 µL of dimethyl sulfoxide (DMSO) was added to each well and provided to dissolve formazan crystals produced by living cells by shaking gently the plate. The optical density (OD) values at 570 nm (as a test wavelength) and 630 nm (as a reference wavelength) were determined using a Rel Assay Diagnostics, BK-EL10C microplate reader (Mega Tıp, Gaziantep, Türkiye). The cytotoxic activity of PSEO was expressed as the cell death rate (%control) and the cell death rate was determined using the following formula.

Cell death (%control) = [1 − (OD_sample_/OD_control_)] × 100

The results were given as mean ± SD of independent experiments. In the analysis, the death rate of untreated control cells was considered to be 0%. To calculate IC_50_ values of PSEO at different incubation times (24, 48, and 72 h), a non-linear regression graph was drawn between %cell death and Log_10_ concentration. Then, IC_50_ values were determined using AAT Bioquest, Inc. Quest Graph™ IC_50_ Calculator (v.1, Sunnyvale, CA, USA). For mechanistic analyses, all biochemical assays (ROS, TAS, caspase-3, caspase-9, Bcl-2, and Bax) were performed at the IC_50_ concentration determined from the 24-h MTT cytotoxicity assay.

### 4.11. Intracellular Reactive Oxygen Species (ROS) Measurement

Intracellular ROS levels were determined using the fluorescent probe 2′,7′-dichlorodihydrofluorescein diacetate (DCFH-DA; Sigma-Aldrich, St. Louis, MO, USA) in U-87MG glioblastoma and SH-SY5Y neuroblastoma cell lines. After the 24 h treatment period, the culture medium was removed, the cells were washed with PBS, and harvested using 0.25% trypsin (Sigma-Aldrich). The cell suspensions were centrifuged and washed twice with PBS. Subsequently, 1 × 10^4^ cells per well were transferred into 96-well plates, and 20 µM DCFH-DA prepared in serum-free medium was added to each well. The cells were incubated with the probe at 37 °C for 30 min in the dark, followed by three washes with PBS to remove excess dye. Fluorescence was measured using a microplate reader on a Perkin-Elmer LS-55 spectrofluorometer at 485 nm excitation and 525 nm emission (5 nm bandwidth for both). Intracellular ROS levels were quantified by comparing the fluorescence intensity of PSEO-treated cells with that of the untreated control group. Values were normalized to the fluorescence of the control and expressed as a percentage of control (% control) [56,57]. The calculation was performed using the following formula.

$$\mathrm{ROS}\;(\%\;\mathrm{of}\;\mathrm{control})=\frac{{\mathrm{Fluorescence}}_{\mathrm{treated}}}{{\mathrm{Fluorescence}}_{\mathrm{control}}}\times 100$$

### 4.12. Total Antioxidant Status (TAS) Analysis

Total antioxidant capacity was quantified using a commercial TAS assay kit (Rel Assay Diagnostics, Cat. No: RL0017; Mega Tıp, Gaziantep, Türkiye) in accordance with the manufacturer’s protocol, with minor procedural adjustments applied. After the 24-h treatment period, the culture media from the control and PSEO (IC_50_ µg/100 µL)-treated U-87MG and SH-SY5Y groups were collected from the 96-well plates and transferred into Eppendorf tubes. Aliquots of 12 μL from each sample, along with Trolox standards and dH_2_O blanks, were pipetted into the corresponding wells of a new 96-well microplate. Next, 200 μL of Reagent 1 was added to all wells, mixed gently, and incubated for 30 s at room temperature in the dark. The first absorbance measurement (A_1_) was recorded at 660 nm. Subsequently, 30 μL of Reagent 2 was added to each well, mixed thoroughly, and allowed to incubate for 10 min under the same conditions. Final absorbance readings (A_2_) were then recorded at 660 nm.

The change in absorbance was calculated as ΔAbs = A_2_ − A_1_. TAS values were computed using the formula provided by the kit and expressed in μmol Trolox equivalent per liter (μmol Trolox Eq./L):

$$\mathrm{TAS}\;(\mathrm{\mu mol}\;\mathrm{Trolox}\;\mathrm{Eq}./L)=(\frac{\Delta {\mathrm{Abs}}_{H_{2}O}-\Delta {\mathrm{Abs}}_{\mathrm{Sample}}}{\Delta {\mathrm{Abs}}_{H_{2}O}-\Delta {\mathrm{Abs}}_{\mathrm{Standard}}})\times 1000$$

### 4.13. Apoptosis-Related Protein Quantification (Caspase-3, Caspase-9, Bcl-2 and Bax) by ELISA Assay

Caspase-3, caspase-9, Bcl-2 and Bax protein levels were quantified in U-87MG and SH-SY5Y cells using human ELISA kits from BT-Lab (Bioassay Technology Laboratory, Shanghai, China) and ThermoFisher Scientific (Waltham, MA, USA). The following kits were used: Human Caspase-3 ELISA Kit (BT-Lab, Cat. No: E4804Hu), Human Caspase-9 ELISA Kit (ThermoFisher, Cat. No: BMS2025), Human Bcl-2 ELISA Kit (BT-Lab, Cat. No: E4035Hu) and Human Bax ELISA Kit (BT-Lab, Cat. No: E4977Hu), following the manufacturer’s instructions. After treatment with PSEO at the IC_50_ concentration for 24 h, cells were washed with cold PBS and detached using trypsin. The collected cell suspensions were adjusted to approximately 1 × 10^6^ cells/mL in PBS (pH 7.2–7.4) and subjected to three freeze–thaw cycles to ensure complete lysis. The lysates were centrifuged at 3000 rpm for 20 min, and the clarified supernatants were collected and stored at −20 °C until analysis.

For ELISA, the supernatants were applied to microplate wells pre-coated with specific antibodies against each target protein. After incubation with streptavidin–HRP conjugate and chromogenic substrate solutions, absorbance was measured at 450 nm using an ELISA microplate reader. Protein concentrations were determined from the respective standard curves and expressed as nanograms per milliliter (ng/mL). In addition, Bcl-2 and Bax values were used to calculate the Bcl-2/Bax ratio as an indicator of the balance between anti-apoptotic and pro-apoptotic signaling.

### 4.14. Statistical Analysis

Statistical analyses were performed using GraphPad Prism software (version 8.0.2, GraphPad Software, San Diego, CA, USA). All data are presented as mean ± standard deviation (SD) from three independent experiments (*n* = 3). Statistical significance was evaluated using two-tailed Student’s *t*-tests when comparing two groups (untreated control vs. IC_50_-treated groups), including ROS, TAS, caspase-3, caspase-9, Bax, Bcl-2, and Bcl-2/Bax ratio analyses. For MTT dose–response experiments, statistical comparisons were conducted using pairwise two-tailed Student’s *t*-tests for each concentration relative to the untreated control group (0 µg/100 µL PSEO), and significance is indicated accordingly in the figures. A *p* value < 0.05 was considered statistically significant.

### 4.15. Artificial Intelligence Use Declaration

During the preparation of this manuscript, the authors used ChatGPT (OpenAI; GPT-5.2) solely for language refinement, clarity improvements, and structural editing of author-drafted text. ChatGPT was not used to generate original scientific content, interpretations, or conclusions. No generative AI tool was used to generate, modify, or fabricate experimental data, to perform statistical analyses, or to determine the scientific conclusions. All AI-assisted edits were reviewed and approved by the authors, who take full responsibility for the integrity and accuracy of this publication.

## 5. Conclusions

Pinus sylvestris essential oil (PSEO) produced measurable, time- and dose-dependent reductions in viability in U-87MG and SH-SY5Y cells and was accompanied by increased intracellular ROS, decreased TAS capacity, activation of caspase-9/caspase-3, Bax upregulation, and Bcl-2 suppression, collectively consistent with a redox-associated intrinsic apoptotic response in vitro.

In parallel, molecular docking was used only as a computational, hypothesis-generating approach to explore whether selected abundant PSEO constituents might plausibly interact with apoptosis-related targets. These in silico predictions suggested feasible binding poses and moderate affinity scores; however, they do not demonstrate direct target engagement or functional modulation and require orthogonal experimental validation (e.g., enzyme inhibition assays, biophysical binding assays).

Overall, the present work should be interpreted as exploratory proof-of-concept evidence restricted to tumor cell models. Further studies should incorporate non-malignant human cell models and selectivity index calculations, standardized oil composition, and in vivo pharmacokinetic/toxicological evaluation before any clinically relevant conclusions can be inferred.

## Figures and Tables

**Table 1:** Phytochemical composition of *Pinus sylvestris* needle essential oil determined by GC–MS analysis. Compounds are listed according to their chromatographic peak no, corresponding to those shown in Figure 1. Retention time, molecular formula, molecular weight, classification and relative percentage of each component are presented. A total of 23 compounds were identified, representing 100% of the volatile profile.

Peak No.	Component Name	Molecular Formula	Molecular Weight (g/mol)	Retention Time (*RT*, min)	% of Total	Classification
1	γ-Terpinene	C_10_H_16_	136.23	4.38	5.15	Monoterpene hydrocarbon
2	p-Cymene	C_10_H_14_	134.22	4.65	4.00	Aromatic compound
3	Bicyclo[3.1.0]hexane, 6-isopropylidene-1-methyl-	C_10_H_16_	136.23	4.96	24.90	Monoterpene hydrocarbon
4	1,2,3,6-Tetrahydrobenzylalcohol, acetate	C_9_H_14_O_2_	154.21	5.30	0.49	Oxygenated monoterpene
5	Benzene, 1-methyl-4-(1-methylethenyl)-	C_10_H_12_	132.20	6.83	1.92	Aromatic compound
6	3-Cyclohexene-1-acetaldehyde, α,4-dimethyl-	C_10_H_16_O	152.23	7.25	1.31	Oxygenated monoterpene
7	Benzene, (2-methoxyethyl)-	C_9_H_12_O	136.19	7.54	0.87	Aromatic compound
8	1,2,4-Methenoazulene, decahydro-1,5,5,8a-tetramethyl-	C_15_H_24_	204.35	7.84	0.58	Sesquiterpene hydrocarbon
9	3-Cyclohexen-1-ol, 1-methyl-4-(1-methylethyl)-	C_10_H_18_O	154.25	8.68	9.81	Oxygenated monoterpene
10	Fenchol	C_10_H_18_O	154.25	8.74	6.67	Oxygenated monoterpene
11	Longifolene	C_15_H_24_	204.35	8.78	1.56	Sesquiterpene hydrocarbon
12	Bicyclo[2.2.1]heptan-2-ol, 2,3,3-trimethyl-	C_10_H_18_O	154.25	8.87	1.02	Oxygenated monoterpene
13	Terpinen-4-ol	C_10_H_18_O	154.25	8.95	4.52	Oxygenated monoterpene
14	Caryophyllene	C_15_H_24_	204.35	9.03	0.65	Sesquiterpene hydrocarbon
15	Cyclohexanol, 1-methyl-4-(1-methylethenyl)-	C_10_H_18_O	154.25	9.23	7.03	Oxygenated monoterpene
16	(1S,3S,4S,5R)-1-Isopropyl-4-methylbicyclo[3.1.0]hexan-3-ol	C_10_H_18_O	154.25	9.63	3.86	Oxygenated monoterpene
17	p-Menth-8-en-1-ol, stereoisomer	C_10_H_18_O	154.25	9.73	1.68	Oxygenated monoterpene
18	α-Terpineol	C_10_H_18_O	154.25	9.95	20.37	Oxygenated monoterpene
19	Bicyclo[3.1.1]hept-2-en-6-ol, 2,7,7-trimethyl-, acetate, [1S-(1α,5α,6β)]-	C_12_H_18_O_2_	194.27	10.17	0.41	Oxygenated monoterpene
20	Naphthalene, 1,2,4a,5,8,8a-hexahydro-4,7-dimethyl-1-(1-methylethyl)-, [1S-(1α,4aβ,8aα)]-	C_15_H_24_	204.35	10.53	0.46	Sesquiterpene hydrocarbon
21	cis-p-Mentha-2,8-dien-1-ol	C_10_H_16_O	152.23	11.07	1.19	Oxygenated monoterpene
22	Benzenemethanol, α,α,4-trimethyl-	C_10_H_14_O	150.22	11.18	1.26	Aromatic compound
23	Ketone, 3abeta,4,5,6,7,7a-hexahydro-7abeta-methyl-1alpha-indanyl methyl	C_12_H_20_O	180.29	11.85	0.28	Non-terpenoid ketone
**Total percentages of compound classes**						
Oxygenated monoterpenes					57.12	
Monoterpene hydrocarbons					38.47	
Sesquiterpene hydrocarbons					3.25	
Aromatic compounds					0.87	
Non-terpenoid ketones					0.29	
**Total**					**100.00**	

**Table 2:** Fatty acid methyl ester (FAME) composition of *Pinus sylvestris* essential oil determined by GC–MS analysis. Compounds are listed according to chromatographic peak numbers (Peak No.) corresponding to those shown in Figure 2. Retention time (RT), molecular formula, molecular weight, and relative percentage (%) are provided for each component. Fifteen FAMEs were identified, accounting for 100% of the total fatty acid composition, with caproic acid (C6:0) and lauric acid (C12:0) representing the predominant constituents.

No.	Peak No.	Component Name	Molecular Formula	Molecular Weight (g/mol)	Retention Time (*RT*, min)	% of Total
1	1	Butyric acid (C4:0)	C_4_H_8_O_2_	88.11	6.55	0.99
2	7	Caproic acid (C6:0)	C_6_H_12_O_2_	116.16	6.95	65.02
3	14	Caprylic acid (C8:0)	C_8_H_16_O_2_	144.21	7.55	0.47
4	25	Capric acid (C10:0)	C_10_H_20_O_2_	172.26	8.62	0.50
5	31	Undecanoic acid (C11:0)	C_11_H_22_O_2_	186.29	9.22	4.98
6	35	Lauric acid (C12:0)	C_12_H_24_O_2_	200.32	10.02	27.33
7	39	Tridecanoic acid (C13:0)	C_13_H_26_O_2_	214.34	10.85	0.04
8	43	Myristic acid (C14:0)	C_14_H_28_O_2_	228.37	12.04	0.13
9	45	Myristoleic acid (C14:1)	C_14_H_26_O_2_	226.35	12.82	0.04
10	46	Cis-10-pentadecenoic acid methyl ester (C15:1)	C_16_H_30_O_2_	254.41	14.43	0.04
11	47	Palmitic acid (C16:0)	C_16_H_32_O_2_	256.42	14.92	0.16
12	48	Heptadecanoic acid (C17:0)	C_17_H_34_O_2_	270.50	16.05	0.03
13	50	Cis-10-heptadecenoic acid (C17:1)	C_17_H_32_O_2_	268.40	17.09	0.05
14	51	Stearic acid (C18:0)	C_18_H_36_O_2_	284.50	17.57	0.03
15	52	Trans-elaidic acid (C18:1)	C_18_H_34_O_2_	282.50	17.95	0.18
**Total**						**100.00**

**Table 3:** Predicted binding affinities of selected ligands against apoptosis-related targets (AutoDock Vina (v1.1.2) implemented in UCSF Chimera v1.17.3; kcal/mol).

Protein	Bicyclo[3.1.0]hexane, 6-Isopropylidene-1-methyl-	α-Terpineol	Caproic Acid (C6:0)
Caspase-9 (1NW9)	−4.8	−5.1	−4.2
Caspase-3 (3KJF)	−4.1	−4.0	−3.4
Bax (4S0O)	−5.4	−5.8	−4.2
Bcl-xL (4C5D)	−5.3	−6.2	−5.0
Endonuclease G (homology model)	−5.7	−3.8	−4.0

## Data Availability

All data generated or analyzed during this study are included in this published article. No additional datasets were created or analyzed. Further inquiries can be directed to the corresponding author.

## Supplementary Materials

The following supporting information can be downloaded at: https://www.mdpi.com/article/10.3390/molecules31030470/s1, Table S1: Raw optical density (OD) values obtained from the MTT assay in U-87MG cells following 24, 48, and 72 h of PSEO treatment; Table S2: Raw optical density (OD) values obtained from the MTT assay in SH-SY5Y cells following 24, 48, and 72 h of PSEO treatment; Table S3: Raw fluorescence intensity values obtained from the intracellular ROS assay in U-87MG and SH-SY5Y cells after 24 h of PSEO treatment; Table S4: Raw absorbance values used for TAS analysis in U-87MG and SH-SY5Y cells after 24 h of PSEO treatment; Table S5: Raw ELISA absorbance values (OD450) and standard curve data for caspase-3 in U-87MG and SH-SY5Y cells after 24 h of PSEO treatment; Table S6: Raw ELISA absorbance values (OD450) and standard curve data for caspase-9 in U-87MG and SH-SY5Y cells after 24 h of PSEO treatment; Table S7: Raw ELISA absorbance values (OD450) and standard curve data for Bax in U-87MG and SH-SY5Y cells after 24 h of PSEO treatment; Table S8: Raw ELISA absorbance values (OD450) and standard curve data for Bcl-2 in U-87MG and SH-SY5Y cells after 24 h of PSEO treatment.

## Abbreviations

The following abbreviations are used in this manuscript:

PSEO	*Pinus sylvestris* Essential Oil
GC–MS	Gas Chromatography–Mass Spectrometry
FAME	Fatty Acid Methyl Ester
IC_50_	Half-Maximal Inhibitory Concentration
ROS	Reactive Oxygen Species
TAS	Total Antioxidant Status
ELISA	Enzyme-Linked Immunosorbent Assay
DCFH-DA	2′,7′-Dichlorodihydrofluorescein Diacetate
DMSO	Dimethyl Sulfoxide
PBS	Phosphate-Buffered Saline
RPMI	Roswell Park Memorial Institute (medium)
SD	Standard Deviation
PDB	Protein Data Bank
SWISS-MODEL	Swiss Homology Modeling Server
UCSF Chimera	University of California San Francisco Molecular Visualization System
Bcl-2	B-cell lymphoma 2 (anti-apoptotic protein)
Bax	Bcl-2-associated X protein (pro-apoptotic protein)
