# Supplementary material for: Chemical Profiling, Molecular Docking, and Mechanistic Anticancer Activity of Pinus sylvestris Essential Oil in SH-SY5Y and U-87MG Cells
Source: Molecules. 2026 Jan 29;31(3):470. doi: 10.3390/molecules31030470 (PMC12898508; doi:10.3390/molecules31030470)
Supplement: Supplementary file 1 [file molecules-31-00470-s001.zip › molecules-4107461-supplementary.pdf]

Supplementary Information (SI) for Molecules.

Supplementary Information

Chemical Profiling, Molecular Docking, and Mechanistic Anticancer Activity of Pinus sylvestris Essential Oil in SH-SY5Y and U-87MG Cells

Gökhan Dervişoğlu [1,*]

Bingöl University, Faculty of Arts and Sciences, Department of Molecular Biology and Genetics, Bingöl, Türkiye;

*Corresponding author

E-mail: gdervisoglu@bingol.edu.tr

Table of Contents

1) **Table S1. Raw optical density (OD) values obtained from the MTT assay in U-87MG cells following 24, 48, and 72 h of PSEO treatment.**

2) **Table S2. Raw optical density (OD) values obtained from the MTT assay in SH-SY5Y cells following 24, 48, and 72 h of PSEO treatment.**

3) **Table S3. Raw fluorescence intensity values obtained from the intracellular ROS assay in U-87MG and SH-SY5Y cells after 24 h of PSEO treatment.**

4) **Table S4. Raw absorbance values used for TAS analysis in U-87MG and SH-SY5Y cells after 24 h of PSEO treatment.**

5) **Table S5. Raw ELISA absorbance values (OD450) and standard curve data for caspase-3 in U-87MG and SH-SY5Y cells after 24 h of PSEO treatment.**

6) **Table S6. Raw ELISA absorbance values (OD450) and standard curve data for caspase-9 in U-87MG and SH-SY5Y cells after 24 h of PSEO treatment.**

7) **Table S7. Raw ELISA absorbance values (OD450) and standard curve data for Bax in U-87MG and SH-SY5Y cells after 24 h of PSEO treatment.**

8) **Table S8. Raw ELISA absorbance values (OD450) and standard curve data for Bcl-2 in U-87MG and SH-SY5Y cells after 24 h of PSEO treatment.**

Table S1. Raw optical density (OD) values obtained from the MTT assay in U-87MG cells following 24, 48, and 72 h of PSEO treatment. Optical density (OD) values were calculated by subtracting the reference wavelength (630 nm) from the test wavelength (570 nm). Each value represents an independent technical replicate (n = 3) per concentration. Data are presented as mean ± standard deviation (SD), calculated using the sample SD formula (n−1).

Time (h)	Concentration (μg/100 μL)	Replicate 1 (OD)	Replicate 2 (OD)	Replicate 3 (OD)	Mean ± SD
24	Control (0)	0.371788314	0.380875	0.389961686	0.380875 ± 0.009087
24	7.81	0.3198	0.3162	0.3193	0.318433 ± 0.001950
24	15.63	0.3256	0.3271	0.3364	0.329700 ± 0.005851
24	31.25	0.3207	0.3192	0.3228	0.320900 ± 0.001808
24	62.5	0.152	0.1508	0.1507	0.151167 ± 0.000723
24	125	0.0858	0.0843	0.0849	0.085000 ± 0.000755
24	250	0.1053	0.1054	0.1049	0.105200 ± 0.000265
24	500	0.1304	0.1312	0.1308	0.130800 ± 0.000400
24	1000	0.094	0.0956	0.0951	0.094900 ± 0.000819
48	Control (0)	0.9545	0.95981	0.964	0.959437 ± 0.004761
48	7.81	0.8748	0.8783	0.8765	0.876533 ± 0.001750
48	15.63	0.8103	0.8173	0.8287	0.818767 ± 0.009287
48	31.25	0.8771	0.894	0.8757	0.882267 ± 0.010185
48	62.5	0.8658	0.8559	0.8523	0.858000 ± 0.006991
48	125	0.2629	0.2693	0.2583	0.263500 ± 0.005524
48	250	0.1421	0.1444	0.1429	0.143133 ± 0.001168
48	500	0.1129	0.1152	0.1153	0.114467 ± 0.001358
48	1000	0.1062	0.1097	0.1052	0.107033 ± 0.002363
72	Control (0)	0.6038	0.6374	0.6709	0.637367 ± 0.033550
72	7.81	0.489	0.454	0.459	0.467333 ± 0.018930
72	15.63	0.511	0.523	0.514	0.516000 ± 0.006245
72	31.25	0.528	0.552	0.519	0.533000 ± 0.017059
72	62.5	0.284	0.278	0.29	0.284000 ± 0.006000
72	125	0.062	0.062	0.068	0.064000 ± 0.003464
72	250	0.077	0.076	0.083	0.078667 ± 0.003786
72	500	0.061	0.057	0.065	0.061000 ± 0.004000
72	1000	0.063	0.061	0.066	0.063333 ± 0.002517

Table S2. Raw optical density (OD) values obtained from the MTT assay in SH-SY5Y cells following 24, 48, and 72 h of PSEO treatment. Optical density (OD) values were calculated by subtracting the reference wavelength (630 nm) from the test wavelength (570 nm). Each value represents an independent technical replicate (n = 3) per concentration. Data are presented as mean ± standard deviation (SD), calculated using the sample SD formula (n−1).

Time (h)	Concentration (μg/100 μL)	Replicate 1 (OD)	Replicate 2 (OD)	Replicate 3 (OD)	Mean ± SD
24	Control (0)	0.4247	0.4359	0.4472	0.435933 ± 0.011250
24	7.81	0.4178	0.4219	0.4199	0.419867 ± 0.002050
24	15.63	0.4859	0.4736	0.4798	0.479767 ± 0.006150
24	31.25	0.4226	0.4236	0.4231	0.423100 ± 0.000500
24	62.5	0.3305	0.3245	0.3275	0.327500 ± 0.003000
24	125	0.0857	0.0841	0.0849	0.084900 ± 0.000800
24	250	0.0791	0.0794	0.0793	0.079267 ± 0.000153
24	500	0.0727	0.0738	0.0733	0.073267 ± 0.000551
24	1000	0.0727	0.0732	0.073	0.072967 ± 0.000252
48	Control (0)	2.1776	2.1903	2.2354	2.201100 ± 0.030376
48	7.81	2.2859	2.2822	2.2819	2.283333 ± 0.002228
48	15.63	1.879	1.8899	1.8785	1.882467 ± 0.006442
48	31.25	1.6826	1.7508	1.6975	1.710300 ± 0.035857
48	62.5	1.3118	1.3045	1.3103	1.308867 ± 0.003855
48	125	0.968	0.9686	0.9529	0.963167 ± 0.008896
48	250	0.1613	0.1676	0.1596	0.162833 ± 0.004215
48	500	0.1138	0.1164	0.1154	0.115200 ± 0.001311
48	1000	0.0645	0.063	0.064	0.063833 ± 0.000764
72	Control (0)	2.6402	2.7036	2.767	2.703600 ± 0.063400
72	7.81	2.1042	2.1418	2.1275	2.124500 ± 0.018979
72	15.63	1.8358	1.833	1.8417	1.836833 ± 0.004441
72	31.25	1.7145	1.7095	1.7123	1.712100 ± 0.002506
72	62.5	1.5169	1.5285	1.4872	1.510867 ± 0.021301
72	125	1.1316	1.1853	1.1658	1.160900 ± 0.027183
72	250	0.5092	0.4958	0.4908	0.498600 ± 0.009514
72	500	0.1084	0.111	0.1095	0.109633 ± 0.001305
72	1000	0.0683	0.0674	0.0701	0.068600 ± 0.001375

Table S3. Raw fluorescence intensity values obtained from the intracellular ROS assay in U-87MG and SH-SY5Y cells after 24 h of PSEO treatment. Intracellular ROS levels were measured using the DCFH-DA fluorescent probe. Fluorescence was recorded at 485 nm excitation and 525 nm emission. Each value represents an independent technical replicate (n = 3). ROS levels were normalized to the untreated control and expressed as percentage of control according to the formula: ROS (% of control) = (Fluorescence treated / Fluorescence control) × 100.

Cell line	Group	Concentration (μg/100 μL)	Replicate 1	Replicate 2	Replicate 3	Mean ± SD	ROS (% of control)
U-87MG	Control	0	0.178	0.172	0.174	0.174667 ± 0.003055	100.00
	PSEO (IC50)	47.93	0.222	0.225	0.221	0.222667 ± 0.002082	127.48
SH-SY5Y	Control	0	0.183	0.183	0.180	0.182000 ± 0.001732	100.00
	PSEO (IC50)	71.63	0.216	0.213	0.218	0.215667 ± 0.002517	118.50

Table S4. Raw absorbance values used for TAS analysis in U-87MG and SH-SY5Y cells after 24 h of PSEO treatment. Total antioxidant status (TAS) was determined using a commercial TAS assay kit. Absorbance was measured at 660 nm before (A1) and after (A2) addition of Reagent 2. The change in absorbance was calculated as ΔAbs = A2 − A1. TAS values were calculated according to the manufacturer's formula and expressed as μmol Trolox equivalent per liter.

Cell line	Group	A1 (660 nm)	A2 (660 nm)	ΔAbs (A2 − A1) – Mean	TAS (μmol Trolox Eq./L)
U-87MG	Control (0 μg/100 μL)	0.0408 / 0.0402 / 0.0407 / 0.0402	0.637 / 0.637 / 0.638 / 0.639	0.597275	172.98
	PSEO (IC50 = 47.93 μg/100 μL)	0.0493 / 0.0496 / 0.0494 / 0.0499	0.654 / 0.653 / 0.655 / 0.657	0.605200	121.04
SH-SY5Y	Control (0 μg/100 μL)	0.042 / 0.0434 / 0.0433 / 0.0434	0.636 / 0.635 / 0.634 / 0.636	0.592225	206.08
	PSEO (IC50 = 71.63 μg/100 μL)	0.0547 / 0.0559 / 0.0559 / 0.0551	0.65 / 0.653 / 0.654 / 0.65	0.596350	179.05
-	dH2O (Blank)	0.0337 / 0.0339 / 0.0348	0.6697 / 0.6662 / 0.6375	0.623667	—
	Standard (1 mmol Trolox/L)	0.0434 / 0.0453 / 0.0463	0.5257 / 0.5125 / 0.5101	0.471100	—

Table S5. Raw ELISA absorbance values (OD450) and standard curve data for caspase-3 in U-87MG and SH-SY5Y cells after 24 h of PSEO treatment. Caspase-3 protein levels were quantified using a commercial human ELISA kit. Absorbance was measured at 450 nm. Standard curve absorbance values correspond to the supplied calibrators and were used to calculate protein concentrations according to the manufacturer's instructions. Each value represents an independent technical replicate.

Section	Cell line	Group / Standard (ng/mL)	Replicate 1	Replicate 2	Replicate 3	Replicate 4
Samples	U-87MG	Control (0 μg/100 μL)	0.6253	0.6468	0.6039	0.6254
	U-87MG	PSEO IC50 (47.93 μg/100 μL)	0.9595	0.8547	0.9110	0.8925
	SH-SY5Y	Control (0 μg/100 μL)	0.6880	0.6868	0.6884	0.6610
	SH-SY5Y	PSEO IC50 (71.63 μg/100 μL)	0.8019	0.7445	0.8379	0.8043
Standards	-	0	0.1185	0.0839	0.1049	-
	-	0.75	0.2244	0.2149	0.2623	-
	-	1.5	0.2945	0.2860	0.3402	-
	-	3	0.4105	0.3895	0.3913	-
	-	6	0.6675	0.6549	0.7386	-
	-	12	1.1495	1.3267	1.4120	-
	-	24	1.9346	2.0882	2.2674	-

Table S6. Raw ELISA absorbance values (OD450) and standard curve data for caspase-9 in U-87MG and SH-SY5Y cells after 24 h of PSEO treatment. Caspase-9 protein levels were quantified using a commercial human ELISA kit. Absorbance was measured at 450 nm. Standard curve absorbance values correspond to the supplied calibrators and were used to calculate protein concentrations according to the manufacturer's instructions. Each value represents an independent technical replicate.

Section	Cell line	Group / Standard (ng/mL)	Replicate 1	Replicate 2	Replicate 3	Replicate 4
Samples	U-87MG	Control (0 µg/100 µL)	0.635	0.643	0.627	0.648
	U-87MG	PSEO IC50 (47.93 µg/100 µL)	0.926	0.918	0.925	0.936
	SH-SY5Y	Control (0 µg/100 µL)	0.68	0.685	0.689	0.676
	SH-SY5Y	PSEO IC50 (71.63 µg/100 µL)	0.83	0.795	0.816	0.821
Standards	-	0	0.085	0.086	0.0855	-
	-	1.6	0.135	0.137	0.136	-
	-	3.1	0.166	0.168	0.167	-
	-	6.3	0.247	0.235	0.241	-
	-	12.5	0.382	0.376	0.379	-
	-	25	0.686	0.700	0.693	-
	-	50	1.139	1.134	1.1365	-
	-	100	2.156	2.163	2.1595	-

Table S7. Raw ELISA absorbance values (OD450) and standard curve data for Bax in U-87MG and SH-SY5Y cells after 24 h of PSEO treatment. Bax protein levels were quantified using a commercial human ELISA kit. Absorbance was measured at 450 nm. Standard curve absorbance values correspond to the supplied calibrators and were used to calculate protein concentrations according to the manufacturer's instructions. Each value represents an independent technical replicate.

Section	Cell line	Group / Standard (ng/mL)	Replicate 1	Replicate 2	Replicate 3	Replicate 4
Samples	U-87MG	Control (0 µg/100 µL)	0.6002	0.6115	0.6039	0.6136
	U-87MG	PSEO IC50 (47.93 µg/100 µL)	0.9595	0.9547	0.9510	0.9425
	SH-SY5Y	Control (0 µg/100 µL)	0.5502	0.5315	0.5339	0.5896
	SH-SY5Y	PSEO IC50 (71.63 µg/100 µL)	0.7319	0.7445	0.7379	0.7343
Standards	-	0	0.047	0.040	0.0435	-
	-	5	0.230	0.227	0.2285	-
	-	10	0.527	0.505	0.516	-
	-	20	0.670	0.674	0.672	-
	-	40	1.242	1.250	1.246	-
	-	80	2.198	2.222	2.210	-

Table S8. Raw ELISA absorbance values (OD450) and standard curve data for Bcl-2 in U-87MG and SH-SY5Y cells after 24 h of PSEO treatment. Bcl-2 protein levels were quantified using a commercial human ELISA kit. Absorbance was measured at 450 nm. Standard curve absorbance values correspond to the supplied calibrators and were used to calculate protein concentrations according to the manufacturer's instructions. Each value represents an independent technical replicate.

Section	Cell line	Group / Standard (ng/mL)	Replicate 1	Replicate 2	Replicate 3	Replicate 4
Samples	U-87MG	Control (0 µg/100 µL)	0.6000	0.6150	0.6150	0.6180
	U-87MG	PSEO IC50 (47.93 µg/100 µL)	0.4602	0.4553	0.4606	0.4592
	SH-SY5Y	Control (0 µg/100 µL)	0.5480	0.5570	0.5610	0.5550
	SH-SY5Y	PSEO IC50 (71.63 µg/100 µL)	0.4610	0.4656	0.4700	0.4656
Standards	-	0	0.0550	0.0460	0.0505	-
	-	3	0.2250	0.2360	0.2305	-
	-	6	0.4560	0.4600	0.4580	-
	-	12	0.7640	0.7740	0.7690	-
	-	24	1.2000	1.3000	1.2500	-
	-	48	2.2960	2.3000	2.2980	-
